# Large Rabi Splitting Driven by Plasmon‐Enhanced Hybrid Anapole States in Si–Au–WSe_2_ Heterostructures

**DOI:** 10.1002/nap2.70296

**Published:** 2026-09-22

**Authors:** Yanhui Deng, Mingyang Yu, Xizhen Xing, Chenxi Zhao, Tianyu Yang, Yujie Ma, Zhang‐Kai Zhou

**Affiliations:** ^1^ Shenzhen Key Laboratory of Ultraintense Laser and Advanced Material Technology, Center for Intense Laser Application Technology, and College of Engineering Physics Shenzhen Technology University Shenzhen China; ^2^ Guangdong Engineering Technology Research Center for High‐Power Ultrafast Laser and X‐Ray Equipment Shenzhen China; ^3^ State Key Laboratory of Optoelectronic Materials and Technologies, School of Physics Sun Yat‐Sen University Guangzhou China; ^4^ Quantum Science Center of Guangdong–Hong Kong–Macao Greater Bay Area (Guangdong) Shenzhen China

**Keywords:** exciton−polariton, hybrid anapole state, metal‐dielectric cavity, plasmon resonance, Rabi splitting

## Abstract

Exciton–polaritons arising from strong light–matter coupling are key enablers of quantum coherent phenomena, whereas large Rabi splitting is critical for ensuring their robustness and stability. However, existing microcavity photonic modes rarely combine low radiative dissipation with strong field confinement, thereby limiting large splitting strong coupling, particularly in the few‐exciton regime. Here, we design a metal–dielectric hybrid cavity in which coherent hybridization among plasmon resonances, magnetic quadrupole modes, and anapole states gives rise to a plasmon‐enhanced hybrid anapole state (HAS) featuring narrow linewidths (54 meV) and strong field localization (180‐fold). Based on the plasmon‐enhanced HAS, we realize both HAS–exciton and HAS–plasmon polaritons and further demonstrate HAS–plasmon–exciton triplex polaritons enabled by multichannel radiative coupling. The ternary system achieves a Rabi splitting of 330 meV, six times larger than the binary case, with a reduced effective exciton number of 6205 based on a characteristic excitonic volume, significantly lower than values reported for the dielectric‐exciton systems under the same volume‐based estimation procedure. A three‐coupled oscillator model elucidates the light–matter interaction mechanism, providing a new pathway to efficient room‐temperature strong coupling and a design guide for high‐performance dielectric‐cavity‐based polariton devices.

## Graphical abstract

A silicon hybrid‐anapole microcavity coupled with a plasmonic array enables tripartite strong coupling with WSe_2_ excitons. Spatially extended field enhancement and coherent cavity–plasmon hybridization strengthen light–matter interactions, producing a giant 330 meV Rabi splitting at room temperature and offering a compact platform for low‐exciton‐density polaritonic photonic devices and applications.

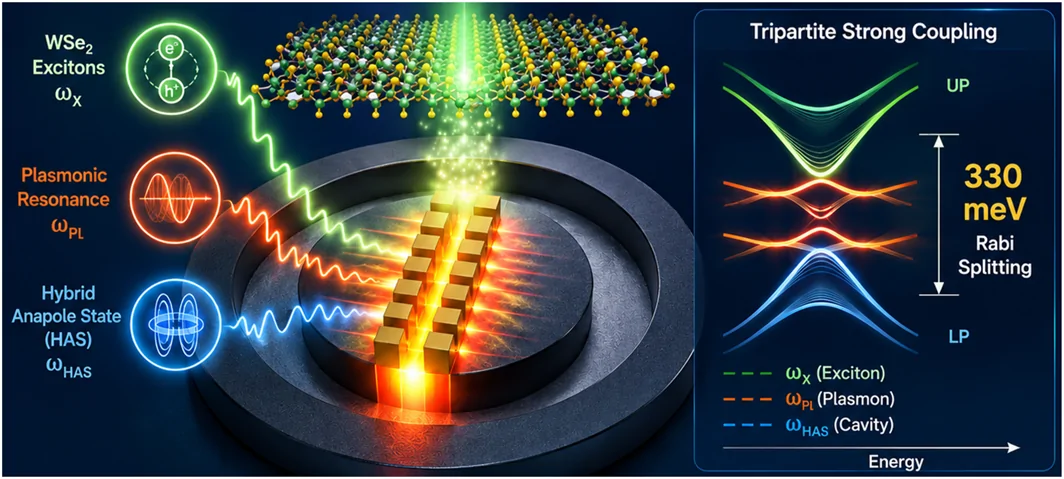

## 1 Introduction

Strong light–matter coupling is central to many modern research and applications, including quantum electrodynamics (QED), ultrafast optics, and topological optics [1, 2, 3]. This regime is when the coherent energy exchange rate between the photonic mode and material excitation (exciton) exceeds their respective decay rates, forming hybrid light–matter quasiparticles (polaritons) [4, 5, 6]. Such couplings hold great potential for quantum manipulation, Bose–Einstein condensation [7, 8], ultrafast optical switching [9, 10, 11], non‐Hermitian photonics [12, 13], and topological polariton devices [14, 15].

In the strong coupling regime, the collective coupling strength for an ensemble of *N* excitons is given by *g =* $\sqrt{N}$ *g*_0_, where *g*_0_ = ***μ*·*E*** is the coupling strength between a single‐exciton and the cavity mode, ***μ*** the exciton transition dipole moment, and ***E*** the electric field of the cavity mode [16, 17]. Enhancing *g* thus requires increasing and aligning ***μ*** and ***E***. Two‐dimensional transition metal dichalcogenides (TMDs) are ideal candidates due to their sharp excitonic resonances and large binding energies that support strongly confined energetic excitons (∼1 nm Bohr radius) [18, 19, 20]. Embedding TMDs layers within optical microcavities enables the realization of strong light–matter coupling, giving rise to polariton eigenstates. This creates opportunities for engineering the exciton–photon interactions.

Plasmonic nanocavities possess strong electric field confinement capabilities and have become representative systems for early room‐temperature strong coupling. Extensive studies have investigated TMDs–plasmonic hybrid systems; however, the intrinsically large dissipation of plasmonic resonances imposes stringent conditions for achieving strong coupling in the visible regime [21, 22, 23, 24, 25], requiring sufficiently large coupling strengths *g* to overcome the coupling threshold *g* > $\sqrt{\left(\gamma^{2}+{\gamma_{\text{ex}}}^{2}\right)/8}$, where *γ* and *γ*_ex_ denote the dissipation rates of the cavity and excitonic modes, respectively. Recently, Liang et al. proposed a local‐to‐nonlocal transition (LNT) strategy to realize high‐quality (high‐*Q*) plasmonic resonances from the near‐to mid‐infrared regime [26], offering a potential route to reduce the strong coupling threshold. Nevertheless, because the maximum achievable *Q* factor is strongly wavelength‐dependent, realizing low‐loss plasmonic modes in the visible range remains challenging. Moreover, purely metallic systems suffer from low damage thresholds and high fabrication costs. Consequently, all‐dielectric and metal–dielectric hybrid platforms have increasingly emerged as promising candidates for realizing room‐temperature strong coupling quantum states. Owing to their high refractive indices, dielectric cavities can support high‐*Q* photonic states with suppressed radiative dissipation through various mechanisms, including surface lattice resonances [27], quasi‐bound states in the continuum (q‐BICs) [7, 28, 29], anapole states [30, 31, 32, 33, 34], and plasmonic–dielectric hybrid states [35, 36, 37, 38], thereby offering opportunities to relax the critical conditions for strong coupling and enhance cavity–exciton interactions. Notably, anapole states are uniquely suited for this application as their far‐field radiation is suppressed by destructive interference between electric dipole (ED) and toroidal dipole (TD) radiation channels, whereas intense electromagnetic energy remains tightly confined inside dielectric nanostructures. Representative investigations on anapole–exciton coupling, higher‐order anapole–exciton coupling, and cavity‐assisted anapole–exciton coupling have demonstrated that spatial and spectral feature of anapole states can effectively tailor exciton–photon interactions [34, 39, 40]. However, achieving both low dissipation and strong electric field confinement within existing photonic modes remains challenging. In particular, the electric field enhancement of high‐*Q* modes is typically limited to below two orders of magnitude, which generally necessitates higher exciton densities and restricts the achievable coupling strengths to only several tens of meV [41, 42, 43, 44].

To address the above challenges, we propose a novel metal–dielectric nanocavity in which the plasmonic resonance hybridizes with the anapole‐associated photonic state, whose optical‐field characteristics originate from the coupled magnetic quadrupole (MQ) and anapole responses, thereby forming a plasmon‐enhanced hybrid anapole state (HAS), achieving an exceptional balance between low dissipation (54 meV) and strong electric field enhancement (180‐fold). The structure can achieve room‐temperature Rabi splittings of 50 and 80 meV for HAS–exciton and HAS–plasmon couplings, respectively. When a plasmonic coupling channel is further introduced, the HAS, plasmon, and exciton form a tripartite coupled system. The resulting UP–LP splitting reaches approximately 330 meV, nearly six times that of the corresponding HAS–exciton system. The enhancement originates from the cooperative contribution of the additional plasmonic coupling channel and the enhanced local field of the hybrid cavity. The effective exciton numbers participating in the HAS–exciton and tripartite HAS–plasmon‐exciton systems are estimated to be only ∼125 and ∼6200, respectively, based on their characteristic excitonic volumes and the TMDs exciton Bohr radii. This mechanism provides an alternative route for achieving large light–matter interaction strengths without relying solely on increasing the exciton population, which is significantly superior to the results reported recently [35, 37, 45] under the same volume‐based estimation procedure. It thus presents a novel platform for designing ultracompact quantum photonic devices.

## 2 Results and Discussions

### 2.1 Design Principal: The Plasmon‐Enhanced HASs in Metal–Dielectric Nanocavities

According to the theoretical expression of the Rabi splitting energy under zero‐detuning conditions, that is, $\Omega =\sqrt{4g^{2}-{\left(\gamma -\gamma_{\text{ex}}\right)}^{2}/4}$ achieving large Rabi splitting requires simultaneously enhancing the coupling strength (*g* ∝ *E*) and suppressing the radiative dissipation of the cavity mode [46]. To this end, we employ the three‐step strategy illustrated in Figure 1a to simultaneously engineer *E* and *γ* in a metal–dielectric nanostructure, including resonance coupling, field pulling, and field amplification. In the first step, under normal incidence of linearly polarized light, a magnetic quadrupole (MQ) and an anapole state are simultaneously excited in the slotted silicon disk–ring structure (System I). Their coherent interference gives rise to a MQ‐anapole state (MAS), which can be compatible with both small *γ* and large *E* to a certain extent. The disk radius is *R*_D_ = 230 nm, the inner and outer ring radii are *R*_in_ = 390 nm and *R*_out_ = 490 nm, and the height of the ring and disk is *H* = 50 nm. The air slot's length and width are *L*_1_ = 260 nm and *d* = 10 nm, respectively. The MAS enables spatially uniform *E* enhancement (> 60 fold), distributed in the air slot of the all‐dielectric SDR (see Figure 1b‐I). However, the enhanced *E* remains predominantly confined inside the cavity and is difficult to efficiently extend into the free‐space region near the cavity surface.

**FIGURE 1:**
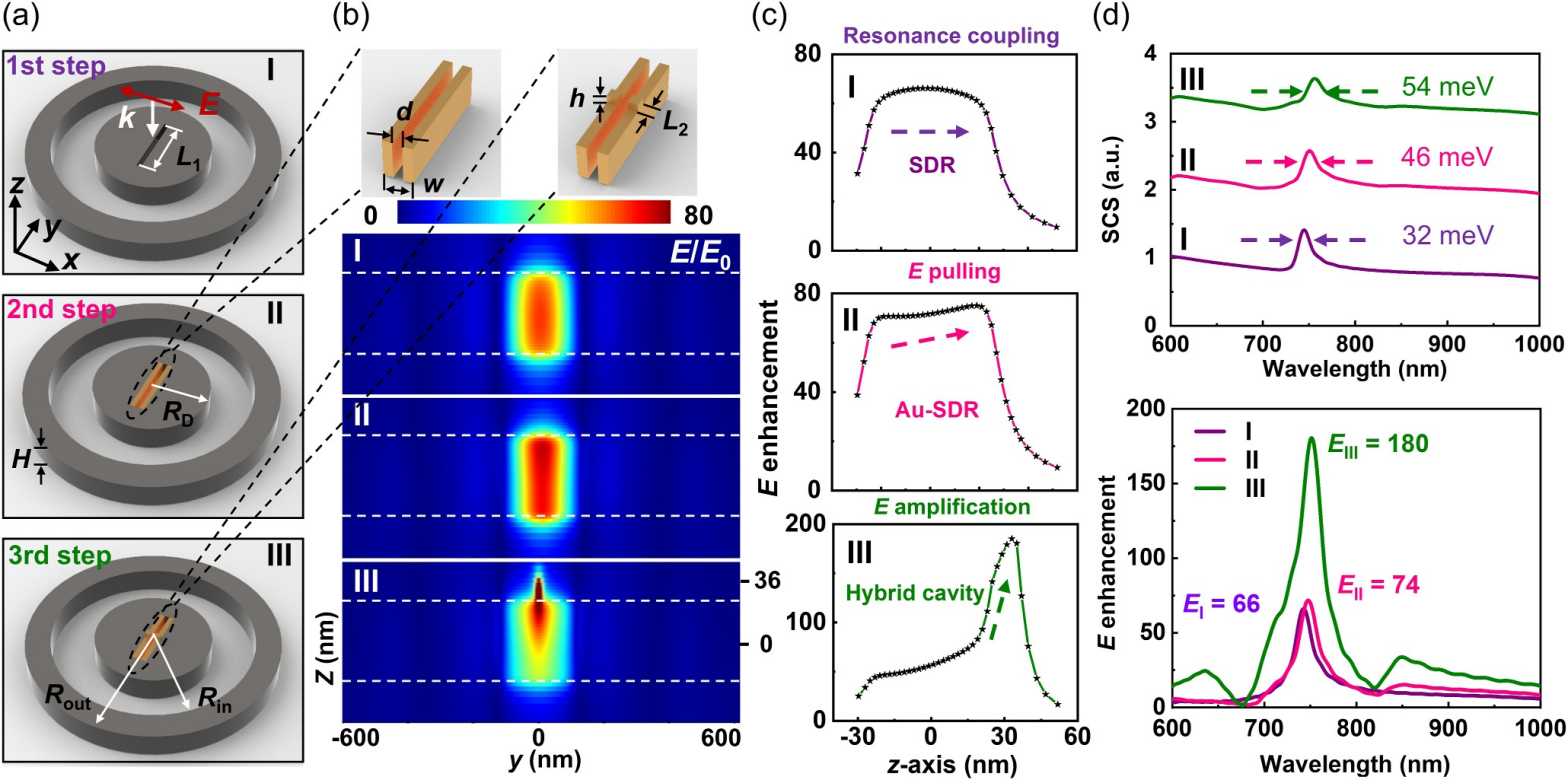
Schematics of the three types of metal–dielectric nanocavities. The origin of the coordinate system is at the center of the disk‐ring structure, with *z* = 0 at the half height of the disk‐ring. In panel (a) and the top part of panel (b): (I) The SDR, a silicon disk‐ring nanocavity with an air slot along the *y* axis. (II) The Au‐SDR, where gold nanoslabs are attached to the inner walls of the air slot. (III) The hybrid cavity, where the gold nanoslabs in II have hat‐like extrusions at their centers as shown in the top‐right panel of (b). Here, *R*_D_ = 230 nm, *R*_in_ = 390 nm, *R*_out_ = 490 nm, *L*_1_ = 260 nm, *w* = 50 nm, *d* = 10 nm, *H* = 50 nm, and *L*_2_ = *h* = 10 nm. The width of the gold nanoslabs is thus (*w*–*d*)/2 = 20 nm. Lower part of panel (b): The resonant near‐field profiles of the nanocavities I, II, and III in the *y–z* plane, with the white dashed lines showing the upper and lower surfaces of the disk‐ring. (c) The electric field (EF, i.e., *E*) enhancement *E*/*E*_0_ along *z* around *y* = 0 in the cavities of three systems. The dashed arrows indicate the trend of the electric field variation. (d) The upper panel shows the simulated scattering cross‐sections (SCS). The resonance linewidths (full‐widths at half‐maximum) are indicated. The lower panel is for the electric field enhsancement spectra of the three cases.

In the second step, we introduced plasmonic resonances by symmetrically embedding a pair of thin (of 20 nm width) gold (Au) slabs along the long edges of the air slot (the Au‐SDR or System II; as in Figure 1a‐II inset). This configuration enhances the uniform *E* by approximately 70‐fold (Figure 1b‐II), approaching the enhancement level achievable in purely plasmonic resonances [16], while effectively pulling the strong *E* toward the structure surface. However, the large mode volume of dielectric cavities tends to decrease the average field intensity, thereby requiring high exciton densities for strong coupling, which limits the realization of room‐temperature single‐qubit quantum states. In the third step, we integrate short gold slabs (with *L*_2_ = *h* = 10 nm and width 20 nm) atop the Au‐SDR structure (the hybrid cavity or System III, see the inset of Figure 1a‐III). This structure confines the fields to within a subwavelength volume, resulting in extreme local enhancement (Figure 1b‐III). Figure 1c illustrates the spatial evolution of the resonant field enhancement (with the coordinate origin at the SDR center) for three cases, that is, a uniform intracavity field, a surface‐enhanced field, and a strongly localized ultrahigh field, corresponding to systems I, II, and III, respectively. The progressive evolution of the near‐field distribution not only broadens the integration schemes between excitonic materials and cavities but also provides an effective route toward enhancing the coupling strength. The peak enhancement factors show remarkable increase, that is, *E*_I_ = 66, *E*_II_ = 74, and *E*_III_ = 180‐fold (Figure 1d, lower panel). Notably, the hybrid cavity supporting a hybrid anapole state (HAS) can efficiently enhance the *E* as well as suppress *γ* (32, 46, and 54 meV for the systems I, II, and III, respectively. Figure 1d, upper panel). Although metal dissipation still causes damping in the visible region, it is much weaker than in pure metal systems. Therefore, this hybrid cavity can be utilized to lower the threshold conditions for realizing room‐temperature strong coupling and to enhance the Rabi splitting.

We now analyze the optical properties of the hybrid cavity to explore the formation mechanism of HAS. Simulated scattering cross‐sections (SCS) for silicon disk radii *R*_D_ ranging from 210 to 240 nm are shown in Figure 2a. The scattering spectra show that the HAS energy decreases monotonically as *R*_D_ is increased, with nearly constant radiative damping and achieving > 160‐fold resonant field enhancement (see Supporting Information S1: Figure S1). We focus on the hybrid cavity with *R*_D_ = 230 nm (red circle in Figure 2a). Electric field enhancement distributions in the *x*–*y* plane (*z* = 0) cross‐section demonstrate counter‐rotating circular displacement currents at the up‐ and down‐sides of the disk‐ring (left panel, Figure 2b). These generate two magnetic dipoles (MD_1_ and MD_2_) oriented perpendicular to the disk‐ring surface (right panel, Figure 2b), forming parallel toroidal dipole moments (TD_1_ and TD_2_) aligned with the incident polarization direction, indicating the emergence of dual subradiant anapole states. Critically, the superposition of dual anapole states enhances the cavity's field confinement capability, amplifying local electric fields. Simultaneously, the antiparallel MD configuration excites a narrow‐linewidth magnetic quadrupole (MQ) mode that ensures low radiative damping of the HAS.

**FIGURE 2:**
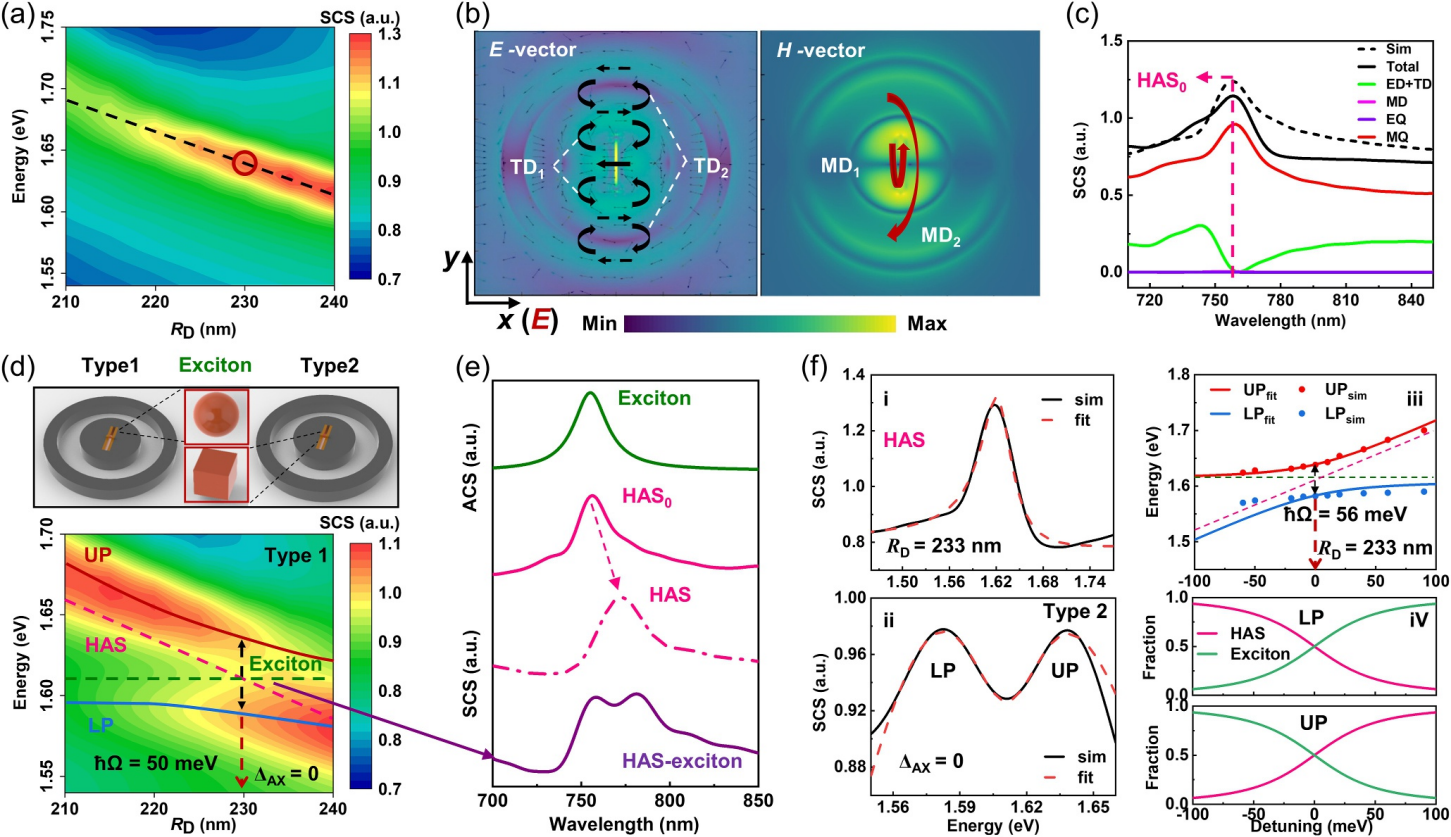
Construction of a two‐level strong coupling system of the HAS–exciton. (a) Scattering cross‐section (SCS) color map with different disk radius *R*_D_ ranging from 210 to 240 nm. (b) Enhanced distribution of electric field (left) and magnetic‐field (right), with arrows indicating the direction of the field vectors. (c) Calculated multipole decompositions of the hybrid cavity exhibited in a SCS plot. ED, TD, MD, EQ, and MQ represent the electric dipole moment, the toroidal dipole moment, the magnetic dipole moment, the electric quadrupole moment, and the magnetic quadrupole moment, respectively. (d) The schematic diagram at the top shows two types of the HAS–exciton coupling systems: the spherical excitons (Type 1, *r* = 5 nm) and the cubic excitons (Type 2, side length = 10 nm), both placed between a pair of gold slabs. The SCS spectra of the HAS–exciton system (Type 1) as a function of disk radius *R*_D_ (in color bar). (e) Simulated resonance curves for Type 1, including the absorption cross‐section (ACS) of the exciton (olive), the intrinsic cavity resonance HAS_0_ without WSe_2_ (magenta), the redshifted bare HAS resonance in the presence of the WSe_2_ background dielectric response (*f* = 0, dashed magenta), and the coupled HAS–exciton response (purple) at the zero‐detuning condition, Δ_AX_ = 0, corresponding to *R*_D_ = 230 nm. (f) Type 2: (i) simulated (solid black curve) and fitted (dashed red curve) scattering spectrum of the HAS‐dominant uncoupled cavity at *R*_D_ = 233 nm and (ii) simulated (solid black curve) and fitted (dashed red curve) scattering spectrum of the corresponding HAS–exciton coupling system at the zero‐detuning condition, Δ_AX_ = 0, corresponding to *R*_D_ = 233 nm.

The dominance of the anapole state and MQ mode in the HAS is further confirmed using multipole expansion analysis (see Supporting Information S1: Section S1). The SCS in Figure 2c demonstrates that the resonance peak (simulation and analysis results, i.e., Sim and Total) is due to the anapole (green curve, resulting from the coupling of ED and TD) [47, 48] and MQ multipoles, accompanied by a minor contribution from the electric octupole (EO, see Supporting Information S1: Figure S2a). Meanwhile, the contributions from the MD and electric quadrupole (EQ) are obscured due to their negligible magnitudes. In particular, the intersection of the oppositely phased ED and TD components near the resonance (see Supporting Information S1: Figure S2b) shows that the anapole dominates the near‐field and the MQ resonance peak dominates the HAS's far‐field radiation, resulting in the characteristic valley‐to‐peak transition.

In low‐dissipation cavity systems, achieving spatial matching between the TMDs positions and the maximum field intensity of the HAS is essential. Two HAS–exciton systems are constructed (Figure 2d): A WSe_2_ sphere (of radius *r* = 5 nm, Type 1) and a WSe_2_ cube (of side length 10 nm, Type 2) are, respectively, embedded in the two gaps between the small gold slabs. The multiphoton quasiparticle coupling in the two hybrid systems is simulated using the finite‐difference time domain (FDTD) method. The WSe_2_‐exciton was treated as an isotropic dielectric with permittivity given by the classical Lorentz oscillator model

(1) $$\varepsilon_{\text{ex}}\left(\omega \right)=\varepsilon_{\infty }+\frac{f\omega_{\text{ex}}^{2}}{{\omega_{\text{ex}}^{2}-\omega }^{2}-i\omega \gamma_{\text{ex}}},$$

where the background permittivity $\varepsilon_{\infty }$ = 18 (high‐energy transitions), the oscillator strength f = 0.4, and the transition energy and radiative damping (full‐widths at half‐maximum, FWHM) of the WSe_2_‐exciton are $\omega_{\text{ex}}$ = 1.64 eV and $\gamma_{\text{ex}}$ = 50 meV, respectively. The parameters are calibrated against experimental data from the references [49, 50]. For exciton polaritons in bulk WSe_2_, setting f = 0.4 yielded the scattering spectra (color bar) versus disk radius *R*_D_ for Type 1 (Figure 2d). Pronounced anticrossing behavior confirms strong coherent HAS–exciton interactions, forming hybridized upper polariton (UP) and lower polariton (LP) branches. Here, the HAS–exciton detuning is defined as $\Delta_{\text{AX}}=\omega_{\text{HA}}-\omega_{\text{ex}}$, where $\omega_{\text{HA}}$ and $\omega_{\text{ex}}$ are the uncoupled HAS and exciton resonance energies, respectively. For Type 1, *R*_D_ = 230 nm corresponds to $\Delta_{\text{AX}}=0$, where the maximum anticrossing occurs. The resulting UP and LP branches exhibit a fitted splitting of approximately 50 meV (Figure 2e). The optically participating WSe_2_ volume *V*_TMD_ of approximately 5.24 × 10^2^ nm^3^, the corresponding effective number of coherently participating excitons *N*_eff_ is estimated to be ∼125 based on the characteristic excitonic volume *V*_0_ ($a_{B}$ ≈ 1 nm is adopted as a representative characteristic Bohr radius for TMD excitons, see Supporting Information S1: Section S4 for details). The absorption cross‐section (ACS) of the WSe_2_‐exciton (olive), together with the SCS of HAS_0_ (magenta solid curve) and HAS (magenta dashed curve), are also presented in Figure 2e. Here, HAS_0_ denotes the intrinsic resonance of the hybrid cavity without WSe_2_. Upon introducing the WSe_2_, its background dielectric response modifies the local refractive‐index environment and induces a redshift of the cavity resonance. We denote this redshifted still uncoupled cavity resonance as HAS (i.e., *f* = 0, bare HAS). The resonance wavelength of the coupled system is redshifted relative to the absorption peak of the WSe_2_‐exciton. In the strong coupling case, the frequency pulling and the energy reorganization of the mixed state would lead to a redshift (relative to the exciton transition and the HAS_0_) of the coupled valleys in the spectral curves. Therefore, we shall use the transition frequency $\omega_{\text{ex}}$ = 1.613 eV of WSe_2_ excitons.

The characteristics of Type 2 are then analyzed to evaluate its capability for achieving room‐temperature strong coupling at low exciton densities (Figure 2f). The corresponding zero‐detuning condition is obtained at *R*_D_ = 233 nm, where the uncoupled HAS‐dominant cavity exhibits a Fano‐like scattering spectrum satisfying $S\left(\omega \right)={\left|s\left(\omega \right)\right|}^{2}$, where [32]

(2) $$s\left(\omega \right)=a_{r}+\sum_{j=1,2}\frac{b_{j}\gamma_{j}e^{i\phi_{j}}}{\omega -\omega_{j}+i\gamma_{j}},$$

and $a_{r}$ is the background amplitude, and $b_{j}$, $\gamma_{j}$, $\phi_{j}$, and $\omega_{j}$ are the amplitude, damping rate, phase, and resonant energy of the oscillator *j*. The fit (red dashed curve in Figure 2f–i) yields a radiative damping rate of $\gamma_{\text{HA}}$ = 54 meV for the HAS. Here, the HAS is described as two coupled modes with largely separated resonance frequencies, namely a bright mode and a dark mode. Since the resonance frequency of the dark mode is far detuned from the excitonic transition frequency, the HAS can be treated as an effective single‐mode cavity predominantly governed by the bright‐mode characteristics [51].

For a quantitative analysis of the HAS–exciton interaction, we use the coupled‐oscillator model [46, 52]:

(3) $$\hbar \left(\begin{array}{cc} \omega_{\text{HA}}-i\frac{\gamma_{\text{HA}}}{2} & g_{\text{AX}}\\ g_{\text{AX}} & \omega_{\text{ex}}-i\frac{\gamma_{\text{ex}}}{2} \end{array}\right)\left(\begin{array}{c} \alpha \\ \beta \end{array}\right)=\hbar \omega \left(\begin{array}{c} \alpha \\ \beta \end{array}\right),$$

where $\omega_{\text{HA}}$ and $\omega_{\text{ex}}$ are the uncoupled‐resonance frequencies of the HAS and the excitons, respectively, $\gamma_{\text{HA}}$ and $\gamma_{\text{ex}}$ are the linewidths of the HAS and the excitons from the FDTD simulation (i.e., the fitted results), and $g_{\text{AX}}$ is the coupling strength between the HAS and the excitons. The eigenvectors *α* and *β* of the UP and LP satisfy ${\left|\alpha \right|}^{2}+{\left|\beta \right|}^{2}=1$. The eigen energies are

(4) $$\hbar \omega_{\pm }=\frac{\hbar \omega_{\text{HA}}+\hbar \omega_{\text{ex}}}{2}-\frac{i\hbar \left(\gamma_{\text{HA}}+\gamma_{\text{ex}}\right)}{4}\pm \frac{\sqrt{4{g_{\text{AX}}}^{2}+{\left(\hbar \omega_{\text{HA}}-\hbar \omega_{\text{ex}}-i\hbar \frac{\gamma_{\text{HA}}}{2}+i\hbar \frac{\gamma_{\text{ex}}}{2}\right)}^{2}}}{2},$$

and the Rabi splitting energy at $\hbar \omega_{\text{HA}}=\hbar \omega_{\text{ex}}$ can be expressed as follows:

(5) $$\hbar \Omega =\hbar \omega_{+}-\hbar \omega_{-}=\hbar \sqrt{{4g_{\text{AX}}}^{2}-\frac{{\left(\gamma_{\text{HA}}-\gamma_{\text{ex}}\right)}^{2}}{4}}.$$

To ensure that the HAS–exciton system be in the strong coupling regime, we must have $g_{\text{AX}}>|\gamma_{\text{ex}}-\gamma_{\text{HA}}|$/4 (so that Rabi splitting occurs) and $g_{\text{AX}}>\sqrt{\left({\gamma_{\text{HA}}}^{2}+{\gamma_{\text{ex}}}^{2}\right)/8}$ (so that Rabi splitting can be experimentally observed) [35]. The coupling strength obtained from the fitting using the SCS expression of the coupled oscillator model under quasi‐static approximation (see Supporting Information S1: Section S2) [51, 53] is 28.02 meV (red dashed curve in Figure 2f‐ii), and Rabi splitting from Equation (5) is 56 meV. Figure 2f‐iii show that by including the coupling strength *g*_AX_ in Equation (3), the fitted dispersions of hybrid states (red solid curve for UP and blue solid curve for LP) agree very well with that (red dots for UP and blue dots for LP) from the simulation. We see that *g*_AX_ satisfies both criteria for strong coupling, consistent with the formation of exciton polaritonic states. Figure 2f‐iv shows that there is significant energy exchange among the HAS and excitonic components in the LP and UP branches.

### 2.2 The HAS–Plasmon Bimodal Strong Coupling Systems

Although strong coupling between HAS‐supported hybrid cavities and excitons has been demonstrated, the coupling strength remains limited. We further propose an enhanced metal‐dielectric hybrid cavity (Figure 3a) comprising an Au‐SDR integrated with 13 pairs of gold slabs to amplify coupling via plasmonic mode hybridization. Compared with the hybrid cavity in Figure 1, the enhanced‐hybrid cavity increases the proportion of the plasmon modes and broadens the area of electric field enhancement, achieving strong coupling between the HAS and plasmon at room temperature. To probe the HAS–plasmon interactions, we systematically tuned the resonance detuning (${\Delta_{\text{AP}}=\omega }_{\text{HA}}-\omega_{\text{pl}}$) while monitoring scattering spectra (Figure 3b). The hybrid UP (red solid curve) and LP (blue solid curve) results in pronounced anticrossing behavior, confirming the formation of HAS–plasmon polaritons. Similarly, the simulated spectrum under the zero‐detuning condition (*R*_D_ = 212 nm) was analyzed using the coupled oscillator model, finding *g*_AP_ = 44.1 meV for the coupling strength between the HAS and the plasmon and 85 meV for the Rabi splitting. The plasmon resonance frequency and radiation damping are $\omega_{\text{pl}}$ = 1.66 meV and $\gamma_{\text{pl}}$ = 101 meV, respectively. The $\omega_{\text{pl}}$ is independently determined from the absorption spectrum of the uncoupled 13‐pair Au‐slab subsystem and then used as the plasmonic reference energy in the SCS and coupled‐oscillator analysis. The strong coupling splitting between the HAS and the plasmon mode is not vanishing and should be experimentally observable. Substituting *g*_AP_, $\omega_{\text{pl}}$, and $\gamma_{\text{pl}}$ for *g*_AX_, $\omega_{\text{ex}}$, and $\gamma_{\text{ex}}$ in Equation (3), we obtain the eigenvalues and Hopfield coefficients versus the energy detuning as shown in Figure 3c,d. The fitted hybrid state dispersions (red/blue solid curves) show excellent agreement with the simulated data (spheres) except in the red‐detuned region (Figure 3c). This minor deviation arises from the high‐energy mode components in the HAS as evidenced by the spectral upturn below 700 nm in the coupled system's SCS (red curve, Figure 3e). The energy exchange process between the HAS (magenta) and the plasmon mode (purple) is also confirmed by the fractions of their components in UP and LP (Figure 3d).

**FIGURE 3:**
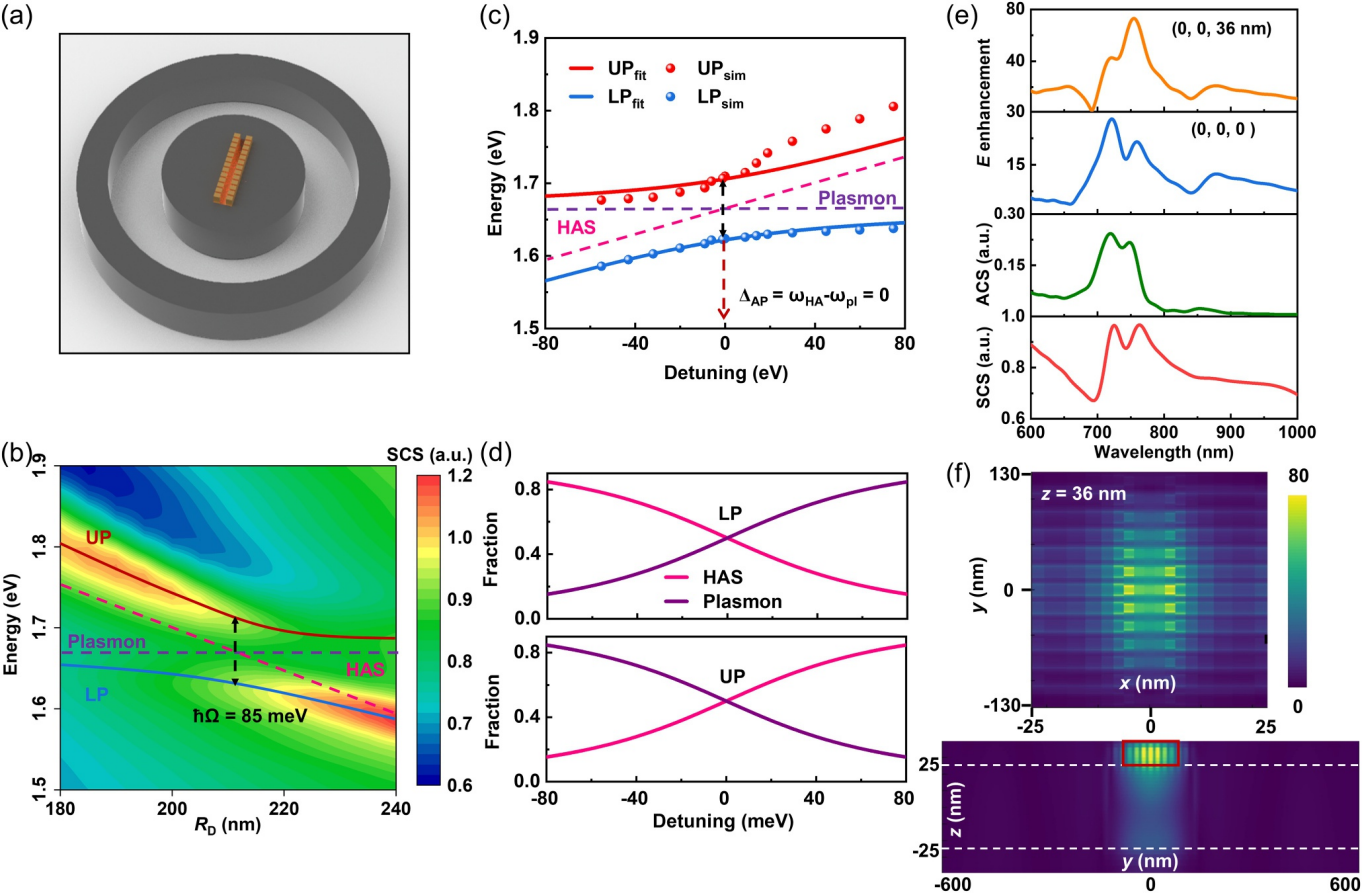
Structure of a two‐level strong coupling system of HAS–plasmons. (a) Schematic of the metal‐dielectric hybrid Au‐SDR cavity and 13 pairs of gold slabs, with the structure dimensions the same as that in Figure 1. (b) The SCS spectra of the HAS–plasmon system as a function of the disk radius *R*_D_ (color bar). (c) The dispersion curves of the strong HAS–plasmon coupling system obtained from the theoretical model (solid curves) and fitting the simulated spectra (spheres). The magenta and purple dashed curves are for the uncoupled HAS and plasmon, respectively. (d) The HAS and plasmon fractions in UP and LP. (e) The SCS (red) and ACS (olive) spectra of the HAS–plasmon system without detuning, that is, *R*_D_ = 212 nm as well as the electric field enhancement spectra at the center (*z* = 0, blue) of the disk‐ring and 1 nm away from the top (*z* = 36 nm, orange) of the hybrid nanocavity. (f) Electric field distributions of the HAS–plasmon system in the *x–y* (*z* = 36 nm) and the *y–z* planes under same color bar.

The physical origins of the enhanced coherent coupling in HAS–plasmon systems are subsequently examined. We quantify the far‐ and near‐field properties of HAS–plasmon polaritons. Figure 3e presents the center point electric field enhancement of the enhanced‐hybrid cavity at the *z* = 0 (blue) and *z* = 36 nm (orange) planes as well as SCS (red) and ACS (olive) at zero detuning. As emphasized in literature [35], concurrent anticrossing in scattering and absorption (or PL) spectra are essential for confirming strong coupling. We confirm the coupling of HAS–plasmon enter the strong coupling regime using the ACS with Rabi splitting and its anticrossing behavior (see Supporting Information S1: Figure S3a). Furthermore, the Rabi splitting behavior in the near‐field spectra at different positions also provides strong evidence for the strong coupling effect of the HAS–plasmon. The corresponding behavior of the electric field enhancement in the *z* = 0 (blue) and *z* = 36 nm (orange) planes is due to the transition of the polarization state of the HAS–plasmon from HAS‐dominant to plasmon‐dominant. Compared to the HAS–exciton system, the polarization state of the HAS–plasmon not only broadens the bandwidth of the local field enhancement but also expands the spatial coverage of high‐field regions. As demonstrated in Figure 3f, the near‐field distributions provide ∼80‐fold enhancement across a 50 × 260 nm^2^ area in the *x–y* (*z* = 36 nm) and *y*–*z* planes. This configuration dramatically increases the possibility of cavity‐exciton alignment, enabling substantial increase of the coupling strength.

### 2.3 Giant Rabi Splitting Induced by the HAS–Plasmon–Exciton Multichannel Coupling

Subsequently, the additional coupling channel is introduced, and a Si−Au−WSe_2_ heterostructure integrating an enhanced hybrid cavity is proposed, in which a 50 × 260 × 1 nm^3^ WSe_2_ film is deposited on the plane at *z* = 36 nm. This configuration facilitates interactions among the hybrid anapole state (HAS), the plasmonic resonance, and the excitonic resonance generate three hybridized polaritonic branches, that is, UP (red solid curve), MP (middle polariton, black solid curve), and LP (blue solid curve) as shown in Figure 4a. While the HAS energy (magenta dashed curve) varies with the silicon disk radius *R*_D_ (210–250 nm), intersecting both plasmon and excitons energies. Since the excitonic contribution to the near‐field distribution of the hybrid cavity is negligible, its influence on the coupling strength between the HAS and plasmon mode can also be ignored. Therefore, the coupling strength of *g*_AP_ is fixed at 44.1 meV in the three‐oscillator system. Applying the coupled mode theory (see Supporting Information S1: Section S3) to simulated scattering spectra yields coupling strengths: *g*_Ax_ (HAS–exciton) = 59.8 meV and *g*_Px_ (plasmon–exciton) = 31.2 meV within this tripartite strong coupling system. The Rabi splitting is computed via an effective 3 × 3 coupled harmonic oscillator model [19, 54]:

(6) $$\hbar \left(\begin{array}{ccc} \omega_{\text{HA}}-i\frac{\gamma_{\text{HA}}}{2} & g_{\text{AP}} & g_{\text{AX}}\\ g_{\text{AP}} & \omega_{\text{pl}}-i\frac{\gamma_{\text{pl}}}{2} & g_{\text{Px}}\\ g_{\text{AX}} & g_{\text{Px}} & \omega_{\text{ex}}-i\frac{\gamma_{\text{ex}}}{2} \end{array}\right)\left(\begin{array}{c} V_{1}\\ V_{2}\\ V_{3} \end{array}\right)=\hbar \omega \left(\begin{array}{c} V_{1}\\ V_{2}\\ V_{3} \end{array}\right),$$

where $V_{1}$, $V_{2}$, and $V_{3}$ are the Hopfield coefficients (satisfying $|V_{1}{|}^{2}+|V_{2}{|}^{2}+|V_{3}{|}^{2}=1$). Figure 4b shows that substituting *g*_AP,_ *g*_Ax_ and *g*_PX_ into Equation (6) results in excellent agreement between the fitted dispersions (solid curves) and simulated data for UP (red squares), ML (black dots), and LP (blue triangles) branches. We define the tripartite Rabi splitting as the UP–LP energy difference (analogous to the bimodal systems). At *R*_D_ = 230 nm, resonant energies of UP (1.651 eV) and LP (1.487 eV) yield ${\hbar \Omega }_{\text{UP}-\text{LP}}$ = 164 meV. The strong coupling criterion for this tripartite system is expressed as follows:

(7) $$\hbar \Omega >W_{\text{UP}}\Gamma_{\text{UP}}+W_{\text{MP}}\Gamma_{\text{MP}}+W_{\text{LP}}\Gamma_{\text{LP}},$$

where, $W_{\text{UP}}$, $W_{\text{MP}}$, and $W_{\text{LP}}$ are the weight coefficients of the UP, MP, and LP states in the HAS–plasmon–exciton heterostructure, and $\Gamma_{\text{UP}}$, $\Gamma_{\text{MP}}$, and $\Gamma_{\text{LP}}$ are the linewidths of the corresponding subsystems. Therefore, the weight contributions of the three branches can be calculated as follows:

(8a) $$W_{\text{UP}}=\Gamma_{\text{UP}}/\left(\Gamma_{\text{UP}}+\Gamma_{\text{MP}}+\Gamma_{\text{LP}}\right),$$

(8b) $$W_{\text{MP}}=\Gamma_{\text{MP}}/\left(\Gamma_{\text{UP}}+\Gamma_{\text{MP}}+\Gamma_{\text{LP}}\right),$$

(8c) $$W_{\text{LP}}=\Gamma_{\text{LP}}/\left(\Gamma_{\text{UP}}+\Gamma_{\text{MP}}+\Gamma_{\text{LP}}\right).$$

**FIGURE 4:**
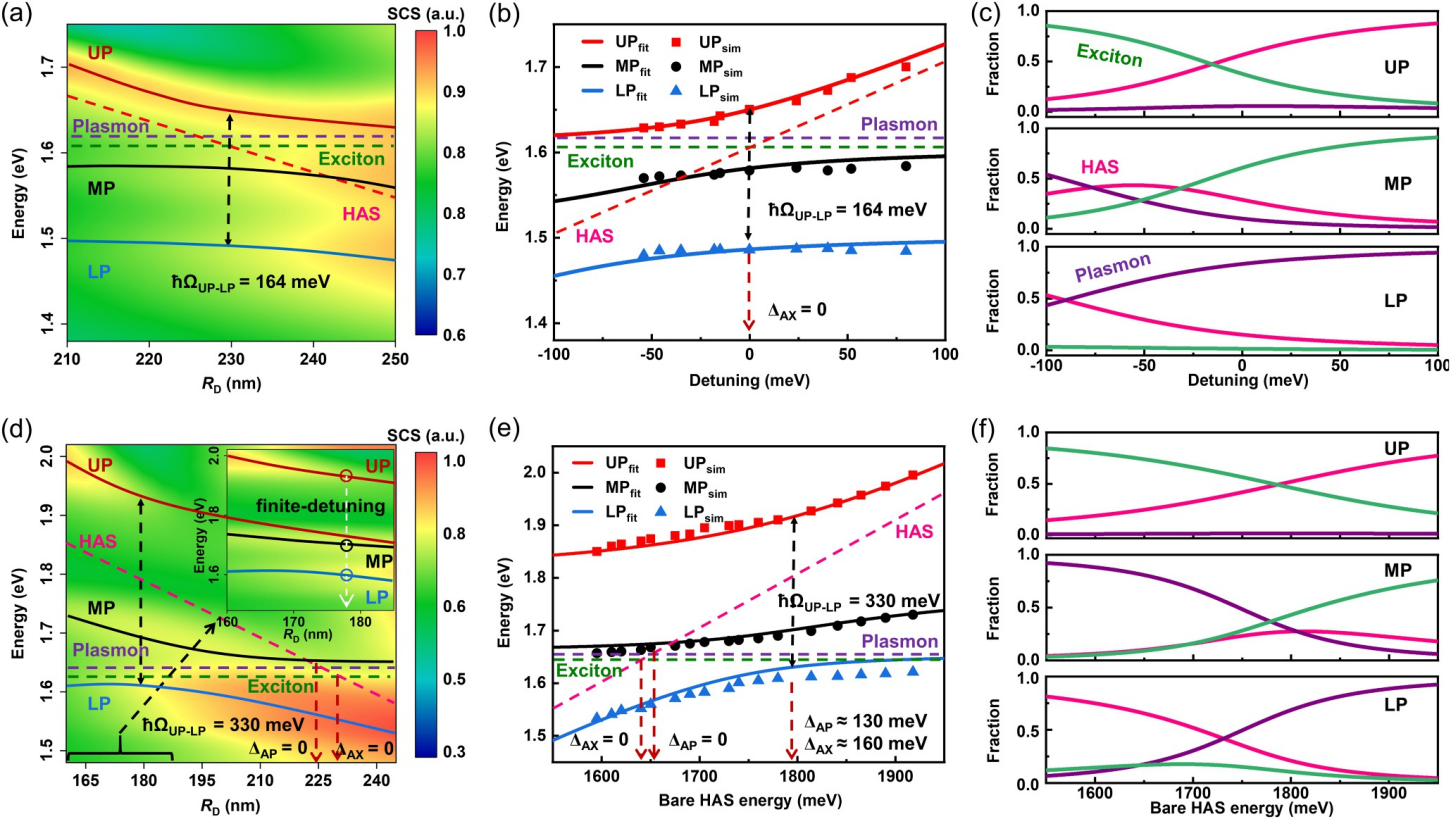
Three‐level strong coupling in the Au‐Si‐WSe_2_ heterostructure. (a–c) A WSe_2_ film with dimensions (*L*_*x*_ × *L*_*y*_ × *L*_*z*_) of 50 × 260 × 1 nm^3^ is located on the upper surface of the nanocavity (*z* = 36 nm). (a) The SCS color map of the HAS–plasmon–exciton system as a function of the disk radius *R*_D_. Three solid curves represent the UP (red), the MP (black), and the LP (blue), respectively. Three dash curves represent HAS resonances (magenta), plasmon resonances (purple), and exciton resonances (olive), respectively. (b) The dispersions curves of the hybrid system obtained by the theoretical model (solid curves) and fitting the simulated spectra (cube, sphere, and triangle). (c) The HAS (magenta), exciton (olive), and plasmon (purple) fractions in UP, MP, and LP. (d–f) A WSe_2_ slab of size 10 × 260 × 10 nm^3^ is placed in the gap area of 13 pairs of gold slabs. (d–f) Same as in panels (a–c), but for the strengthened Si−Au−WSe_2_ heterostructure (see text). The inset in (d) shows the SCS heatmap in the *R*_D_ = 160–185 nm range. Distinct anticrossing behavior can be clearly observed.

Using the Hopfield coefficients in Figure 4c, the linewidths of the generated three branches are

(9a) $$\Gamma_{\text{UP}}=0.565\gamma_{\text{HA}}+0.057\gamma_{\text{pl}}+0.378\gamma_{\text{ex}},$$

(9b) $$\Gamma_{\text{MP}}=0.290\gamma_{\text{HA}}+0.103\gamma_{\text{pl}}+0.607\gamma_{\text{ex}},$$

(9c) $$\Gamma_{\text{LP}}=0.151\gamma_{\text{HA}}+0.834\gamma_{\text{pl}}+0.015\gamma_{\text{ex}}.$$

Substituting Equations ((8a), (8b), (8c)) and ((9a), (9b), (9c)) into Equation (7), the strong coupling criterion for the HAS–plasmon–exciton heterostructure can be obtained as follows:

(10) $$\hbar \Omega >0.3\gamma_{\text{HA}}+0.42\gamma_{\text{pl}}+0.28\gamma_{\text{ex}},$$

where the FWHM values of the HAS, the plasmon, and the excitons are $\gamma_{\text{HA}}$ = 54, $\gamma_{\text{pl}}$ = 101, and $\gamma_{\text{ex}}$ = 50 meV, respectively. Moreover, we have for the Rabi splitting ${\hbar \Omega }_{\text{UP}-\text{LP}}$ = 164 meV. Introducing plasmon–dielectric cavity coupling into the hybrid nanocavity, namely the formation of a plasmon‐enhanced HAS, can significantly strengthen the cavity photon–exciton interaction. Notably, the Rabi splitting observed in the three‐oscillator system (plasmon–HAS–exciton) is substantially larger than that in either of the corresponding two‐oscillator systems, namely HAS–exciton (56 meV) and HAS–plasmon (85 meV). This enhancement arises from the combined effects of increased local field intensity and the additional coupling channel provided by the plasmonic mode. Based on the optically participating WSe_2_ volume of approximately 1.3 × 10^4^ nm^3^, the corresponding effective number of coherently participating excitons *N*_eff_ is estimated to be ∼3.1 × 10^3^.

Finally, to further enhance the spatial overlap between excitons and localized fields (a critical factor governing coupling strength), we designed a strengthened Si−Au−WSe_2_ heterostructure comprising of an enhanced‐hybrid cavity and a 10 × 260 × 10 nm^3^ WSe_2_ bulk placed in the gap region of 13 pairs of gold slabs. As shown in Figure 4d, enhanced interactions among HAS, plasmon, and exciton resonances generate triply hybridized states (UP, MP, and LP) exhibiting a giant Rabi splitting (${\hbar \Omega }_{\text{UP}-\text{LP}}$ ∼330 meV). Additionally, the anticrossing behavior in the ACS spectrum is also demonstrated (see Supporting Information S1: Figure S3b). With *g*_AP_ = 44.1 meV fixed, the coupling strengths of HAS–plasmon and HAS–exciton were fitted to be *g*_AX_ = 145 meV and *g*_PX_ = 40 meV, respectively. Zero‐detuning serves as a critical reference condition for identifying the anticrossing characteristics of conventional two‐resonance strong coupling systems [55, 56]. Different from the two‐resonance strong coupling framework, the enhanced tripartite structure is designed such that the HAS remains at finite detuning from the plasmonic (Δ_AP_ = *ω*_HA_–*ω*_pl_ = 130 meV) and excitonic resonances (Δ_AX_ = *ω*_HA_–*ω*_ex_ = 160 meV) over the specific parameter range (*R*_D_ = 160–250 nm), which in turn reduces the visual prominence of the anticrossing behavior. Nevertheless, the coupling strengths are sufficiently large to produce pronounced three‐resonance hybridization. As a result, the three basis states are strongly hybridized, giving rise to nearly symmetric triple splitting rather than weak perturbative shifts (See the inset in Figure 4d). The SCS map provides the spectral signature of the hybridized resonances, whereas the anticrossing behavior is identified from the polaritonic branches dispersion and further supported by the Hopfield coefficients.

The fitted dispersion curves (solid curve) of the three hybrid states of the current system and the simulation data (UP, red cubes, MP, black spheres, and LP, blue triangles) are presented in Figure 4e, and they show consistency. The detuning‐dependent calculation provides the polaritonic branches evolution of the 3 × 3 Hamiltonian as the bare HAS resonance energy is varied. The zero‐detuning positions (Δ_AP_ = 0 and Δ_AX_ = 0) are used as reference points, whereas the 330 meV configuration is located at finite detuning. By diagonalizing Equation (6), we resolve the composition evolution of HAS, plasmon, and excitons components within UP/MP/LP branches as functions of the bare HAS's resonance energy (Figure 4f).

By combining the Hopfield coefficient with Equations ((7), (8a), (8b), (8c), (9a), (9b), (9c)), we can obtain $\Gamma_{\text{UP}}$ = 71.78 meV, $\Gamma_{\text{MP}}$ = 78.62 meV, and $\Gamma_{\text{LP}}$ = 54.09 meV as well as $W_{\text{UP}}$ = 0.35, $W_{\text{MP}}$ = 0.38, and $W_{\text{LP}}$ = 0.27, and the formal strong coupling criterion for HAS–plasmon–exciton heterostructures as follows:

(11) $$\hbar \Omega >0.35\gamma_{\text{HA}}+0.35\gamma_{\text{pl}}+0.3\gamma_{\text{ex}}.$$

The coupling system satisfies the strong coupling criterion. More significantly, the Rabi splitting magnitude reaches nearly sixfold that of the standalone strong HAS–exciton coupling system, demonstrating that the introduction of the HAS–plasmon strong coupling configuration substantially enhances the coherent interaction between HAS and excitons. Similarly, the estimated *N*_eff_ is approximately 6.2 × 10^3^, which is significantly lower than that reported in other dielectric cavity–exciton strong coupling systems under the same volume‐based estimation procedure. We additionally use $\hbar \Omega /\sqrt{N_{\text{eff}}}$ as a normalized coupling metric to compare the intrinsic coupling efficiency among different cavity–exciton systems. This normalization partially removes the trivial collective enhancement associated with different effective exciton populations and therefore provides a more meaningful comparison of the coupling strength per effective excitonic degree of freedom (see Supporting Information S1: Table S1).

## 3 Conclusion

In this work, we demonstrate that the combination of plasmon resonances and HASs can significantly enhance the electric field confinement capability of optical microcavities while preserving low radiative dissipation of HASs, thereby boosting photon–exciton coupling strength and lowering the strong coupling threshold. Specifically, the proposed HAS–plasmon–exciton hybrid system achieves a giant Rabi splitting of 330 meV. The metal–dielectric hybrid cavity supports up to 180‐fold local electric field enhancement while maintaining a low dissipative damping of 54 meV through plasmon‐enhanced HASs. In addition, the system enables the realization of dual‐branch polaritonic states, including HAS–exciton and HAS–plasmon polaritons as well as ternary HAS–plasmon–exciton polaritons. Based on the characteristic Bohr radius of WSe_2_ excitons, the effective numbers of excitons participating in the coupling are estimated to be only 6205 for the HAS–plasmon–exciton system and 125 for the HAS–exciton system, both significantly lower than those reported in previous dielectric cavity–exciton strong coupling studies under the same volume‐based estimation procedure. These results establish the proposed coupled architecture as a promising platform for exploring strong light–matter interactions and provide a new route toward cavity–multiexciton polaritonic systems in quantum photonics.

## 4 Methods

Finite‐difference time‐domain (FDTD) simulations are performed for far‐field and near‐field calculations. Material parameters for Si, Au, and WSe_2_ are obtained from published datasets. The mesh volumes are configured as follows: 5 × 5 × 5 nm^3^ for the Au‐SDR cavity, 2 × 2 × 2 nm^3^ for the air slot, 1 × 1 × 1 nm^3^ for the WSe_2_ nanosphere/cube, and 4 × 2 × 0.1 nm^3^ for the WSe_2_ monolayer with a simulation domain mesh volume of 4 × 4 × 2 μm^3^. The scattering cross‐sections, absorption cross‐sections, and electric field enhancements are calculated using a total‐field/scattered‐field (TFSF) source spanning 400–1200 nm.

## Author Contributions

**Yanhui Deng:** conceptualization, data curation, formal analysis, investigation, methodology, funding acquisition, project administration, writing – original draft, writing – review and editing. **Mingyang Yu:** investigation, writing – original draft. **Xizhen Xing:** data curation, methodology, investigation. **Chenxi Zhao:** data curation, investigation. **Tianyu Yang:** data curation, investigation. **Yujie Ma:** conceptualization, funding acquisition, project administration, validation, writing – review and editing. **Zhang‐Kai Zhou:** conceptualization, funding acquisition, project administration, supervision, writing – review and editing.

## Conflicts of Interest

The authors declare no conflicts of interest.

## Supporting information


Supporting Information S1

## Data Availability

The data that support the findings of this study are available from the corresponding author upon reasonable request.
