# Supplementary material for: Large Rabi Splitting Driven by Plasmon‐Enhanced Hybrid Anapole States in Si–Au–WSe2 Heterostructures
Source: Nanophotonics. 2026 Sep 22;15(18):e70296. doi: 10.1002/nap2.70296 (PMC13672129; doi:10.1002/nap2.70296)
Supplement: Supplementary file 1 [file NAP2-15-e70296-s001.pdf]

# Supporting Information for:

# Large Rabi Splitting Driven by Plasmon-Enhanced Hybrid Anapole States in Si–Au–$WSe_2$ Heterostructures

*Yanhui Deng*[1], *Mingyang Yu*[1], *Xizhen Xing*[1], *Chenxi Zhao*[1], *Tianyu Yang*[1], *Yujie Ma*[1,2,*], *and Zhang-Kai Zhou*[3,4]

[1]Shenzhen Key Laboratory of Ultraintense Laser and Advanced Material Technology, Center for Intense Laser Application Technology, and College of Engineering Physics, Shenzhen Technology University, Shenzhen 518118, China;

[2]Guangdong Engineering Technology Research Center for High-Power Ultrafast Laser and X-Ray Equipment, Shenzhen 518118, China;

[3]State Key Laboratory of Optoelectronic Materials and Technologies, School of Physics, Sun Yat-sen University, Guangzhou 510275, China;

[4]Quantum Science Center of Guangdong–Hong Kong–Macao Greater Bay Area (Guangdong), Shenzhen 518045, China

*Corresponding Author: mayujie@sztu.edu.cn

## TABLE OF CONTENT

Supporting Notes, Sections S1 to S4

Supporting Figures S1 to S3

Supporting Table S1

References

**Supporting Notes:**

**Section S1. Details for multipole decompositions**

We quantify the contributions of electric dipole (ED), magnetic dipole (MD), electric quadrupole (EQ), and magnetic quadrupole (MQ) moments to the scattering cross section (SCS) of nanostructures using multipole decomposition. The multipole moments are derived from the induced current density $\hat{J}_\omega(\hat{r})$, i.e. [1-3],

ED moment:

$$p_\alpha = -\frac{1}{i\omega}\left\{\int d^3\hat{r}\, J_\alpha^\omega j_0(kr) + \frac{k^2}{2}\int d^3\hat{r}[3(\hat{r}\cdot\hat{J}_\omega)r_\alpha - r^2 J_\alpha^\omega]\frac{j_2(kr)}{(kr)^2}\right\}, \quad (1)$$

It is noteworthy that the expression renders the introduction of toroidal multipoles (i.e., the third family of multipoles) unnecessary. This formulation reveals that the toroidal moment constitutes merely higher-order terms in the electric multipole expansion, implying that the resultant expression inherently incorporates the combined contributions of both the electric and toroidal dipoles (ED+TD) to the scattering cross section.

MD moment:

$$m_\alpha = \frac{3}{2}\int d^3\hat{r}\,(\hat{r}\times\hat{J}_\omega)_\alpha \frac{j_1(kr)}{kr}, \quad (2)$$

EQ moment:

$$Q_{\alpha\beta}^e = -\frac{3}{i\omega}\left\{\int d^3\hat{r}\,[3(r_\beta J_\alpha^\omega + r_\alpha J_\beta^\omega - 2(\hat{r}\cdot\hat{J}_\omega)\delta_{\alpha\beta}]\frac{j_1(kr)}{kr} + 2k^2\int d^3\hat{r}[5r_\alpha r_\beta(\hat{r}\cdot\hat{J}_\omega) - (r_\alpha J_\beta + r_\beta J_\alpha)r^2 + r^2(\hat{r}\cdot\hat{J}_\omega)\delta_{\alpha\beta}]\frac{j_3(kr)}{(kr)^3}\right\}, \quad (3)$$

MQ moment:

$$Q_{\alpha\beta}^m = 15\int d^3\hat{r}\left\{r_\alpha(\hat{r}\times\hat{J}_\omega)_\beta + r_\beta(\hat{r}\times\hat{J}_\omega)_\alpha\right\}\frac{j_2(kr)}{(kr)^2}, \quad (4)$$

where $\alpha$, $\beta \in \{x, y, z\}$ while $\omega$, $k$, $c$, $\hat{r}$ denote angular frequency, wavenumber, speed of light, and position vector, respectively. The induced current density is given by $\hat{J}_\omega(\hat{r}) = i\omega\varepsilon_0(\varepsilon_r - 1)E_\omega(\hat{r})$, where $E_\omega(\hat{r})$ is the electric field distribution, $\varepsilon_0$ the vacuum permittivity, and $\varepsilon_r$ the relative permittivity. Using FDTD simulations, we obtain the normalized electric field distributions $E_\omega(\hat{r})/E_{inc}$, with $E_{inc}$ being the

incident field amplitude. The functions $j_1(kr)$, $j_2(kr)$ and $j_3(kr)$ represent the spherical Bessel functions. The multipole-derived scattering cross section is expressed as:

$$C_{sca}^{total} = C_{sca}^{p} + C_{sca}^{m} + C_{sca}^{Q^e} + C_{sca}^{Q^m} + \cdots$$

$$= \frac{k^4}{6\pi{\varepsilon_0}^2|E_{inc}|^2}\left[\sum_\alpha \left(|p_\alpha|^2 + \frac{|m_\alpha|^2}{c}\right) + \frac{1}{120}\sum_\alpha \left(|kQ_{\alpha\beta}^e|^2 + \left|\frac{kQ_{\alpha\beta}^e}{c}\right|^2\right) + \cdots\right] \quad (5)$$

**Section S2. Coupled mode theory (CMT) of a Fano-like two-mode system**

The coupled-oscillator model provides a classical theoretical framework for describing the interaction between two resonant modes [4,5]. For a system involving the coupling between a Fano-like cavity mode and an exciton resonance, the cavity mode should be regarded as arising from the hybridization of two spectrally separated eigenmodes, each of which can, in principle, interact with the exciton mode. However, since the resonance frequency of the detuned cavity mode is far away from the excitonic transition frequency, the corresponding coupling strength is negligibly small. Therefore, the interaction between the detuned cavity mode and the exciton is neglected in our model. Following the derivation presented in the literature [5], the scattering cross section of the coupled system can be obtained under the quasi-static approximation and a new expression as follows. A more detailed derivation can be found in our previous work [6].

$$s(\omega) \propto A\omega^4\{\left|\frac{(\omega_{ex}^2 - \omega^2 - i\gamma_{ex}\omega)}{(\omega^2 - \omega_1^2 + i\gamma_1\omega)(\omega^2 - \omega_{ex}^2 + i\gamma_{ex}\omega) - 4\omega^2 {g_1}^2}\right|^2 + \left|\frac{(\omega_{ex}^2 - \omega^2 - i\gamma_{ex}\omega)}{(\omega^2 - \omega_2^2 + i\gamma_2\omega)(\omega^2 - \omega_{ex}^2 + i\gamma_{ex}\omega) - 4\omega^2 {g_2}^2}\right|^2 + 2\left|\frac{(\omega_{ex}^2 - \omega^2 - i\gamma_{ex}\omega)^2}{[(\omega^2 - \omega_1^2 + i\gamma_1\omega)(\omega^2 - \omega_{ex}^2 + i\gamma_{ex}\omega) - 4\omega^2 {g_1}^2][(\omega^2 - \omega_2^2 + i\gamma_2\omega)(\omega^2 - \omega_{ex}^2 + i\gamma_{ex}\omega) - 4\omega^2 {g_2}^2]}\right|\} \quad (6)$$

where, $\omega_1$ and $\omega_2$ denote the resonance frequencies of the two far-detuned cavity modes, $\gamma_1$ and $\gamma_2$ represent their respective radiative damping rates, and

$A$ corresponds to the scattering amplitude of the cavity–exciton coupled system.

**Section S3. Coupled mode theory (CMT) of a three-resonance system**

Within the framework of Coupled Mode Theory (CMT), the hybrid anapole state (HAS), the plasmon mode, and the exciton mode are treated as mutually coupled harmonic oscillators. To accurately reproduce the scattering spectra shown in Figure 4, it is necessary to simultaneously consider the pairwise interactions among the hybrid anapole state (HAS), the plasmonic resonance (pl), and the excitonic resonance (ex), characterized by the coupling strengths $g_{\mathrm{AP}}$ (HAS-plasmon), $g_{\mathrm{AX}}$ (HAS-exciton), and $g_{\mathrm{PX}}$ (plasmon-exciton), respectively. Under this formalism, the dynamical response of the hybrid system can be described by the coupled-oscillator matrix ($M$) expressed as [7,8]:

$$M = \begin{pmatrix} \omega_{\mathrm{HA}} - i\frac{\gamma_{\mathrm{HA}}}{2} & g_{\mathrm{AP}} & g_{\mathrm{AX}} \\ g_{\mathrm{AP}} & \omega_{\mathrm{pl}} - i\frac{\gamma_{\mathrm{pl}}}{2} & g_{\mathrm{PX}} \\ g_{\mathrm{AX}} & g_{\mathrm{PX}} & \omega_{\mathrm{ex}} - i\frac{\gamma_{\mathrm{ex}}}{2} \end{pmatrix}, \tag{7}$$

where, $\omega_{\mathrm{ex}}$, $\omega_{\mathrm{pl}}$ and $\omega_{\mathrm{HA}}$ denote the coherent resonance frequencies of the exciton mode, the plasmon mode, and the HAS mode, respectively. The parameters $\gamma_{\mathrm{ex}}$, $\gamma_{\mathrm{pl}}$ and $\gamma_{\mathrm{HA}}$ represent the linewidths of the respective modes. The direct coupling between the plasmon and exciton modes is neglected due to their resonance detuning. Specifically, in the open system considered in this work—comprising a subwavelength Mie resonator coupled to two-dimensional transition metal dichalcogenides (TMDs)—the scattering cross section $s(\omega)$ can be expressed as:

$$s(\omega) \propto A\left|\frac{(\omega-W_{HA})(\omega-W_{ex})-g_{\mathrm{AX}}^2}{(\omega-W_{HA})(\omega-W_{pl})(\omega-W_{ex})+2g_{\mathrm{AX}}g_{\mathrm{AP}}g_{\mathrm{PX}}-(\omega-W_{HA})g_{\mathrm{PX}}^2-(\omega-W_{pl})g_{\mathrm{AX}}^2-(\omega-W_{ex})g_{\mathrm{AP}}^2}\right|^2 + B, \tag{8}$$

where $W_{pl} = \omega_{\mathrm{pl}} - i\gamma_{\mathrm{pl}}/2$, $W_{ex} = \omega_{\mathrm{ex}} - i\gamma_{\mathrm{ex}}/2$ and $W_{HA} = \omega_{\mathrm{HA}} - i\gamma_{\mathrm{HA}}/2$. $A$ and $B$ represent the scattering amplitude and background amplitude of the

coupled system respectively.

## Section S4. Estimation of the effective number of coherently participating excitons

To provide a consistent geometrical metric for comparing the amount of excitonic material involved in the cavity–exciton interaction, we estimate an effective exciton number $N_{\mathrm{eff}}$ from the optically participating TMD volume. For a periodic monolayer TMD, the effective material volume within one structural unit cell is defined as

$$V_{\mathrm{TMD}} = P_{\mathrm{x}} P_{\mathrm{y}} t_{\mathrm{TMD}}, \qquad (9)$$

where $P_{\mathrm{x}}$ and $P_{\mathrm{y}}$ are the structural periods and $t_{\mathrm{TMD}} \approx 1$ nm is the nominal monolayer thickness. For the bulk-TMD configuration, $V_{\mathrm{TMD}}$ is taken as the geometric volume of the TMD region overlapping with the localized optical field. TMD excitons possess a strongly confined two-dimensional character, with a characteristic Bohr radius on the order of 1 nm [9,10]. To construct a consistent volume-based normalization, we define the characteristic excitonic volume as

$$V_0 = \frac{4}{3}\pi a_B^3, \qquad (10)$$

where $a_B \approx 1$ nm. The effective number of coherently participating excitons is then estimated as

$$N_{eff} = \frac{V_{TMD}}{V_0}. \qquad (11)$$

For the first tripartite configuration, $V_{TMD} \approx 1.3 \times 10^4$ nm$^3$, yielding $N_{eff} \approx 3.1 \times 10^3$. For the strengthened heterostructure, the active $WSe_2$ volume is $10 \times 260 \times 10 = 2.6 \times 10^4$ nm$^3$, corresponding to $N_{eff} \approx 6.2 \times 10^3$.

It should be emphasized that $N_{\mathrm{eff}}$ is an effective geometrical estimate rather than a direct calculation of the instantaneous exciton population. It is introduced to quantify and compare the effective amount of excitonic material participating in the optical interaction under a consistent normalization.

Because the collective light–matter coupling generally scales as $g \propto \sqrt{N_{eff}}$, we additionally use $\frac{\hbar\Omega}{\sqrt{N_{eff}}}$ as a normalized coupling metric to compare the intrinsic coupling efficiency among different cavity–exciton systems. This normalization partially removes the trivial collective enhancement associated with different effective exciton populations and therefore provides a more meaningful comparison of the coupling strength per effective excitonic degree of freedom (see Table S1).

**Figure S1. The far- and near-field characteristic spectra of hybrid cavities with different disk radii.**

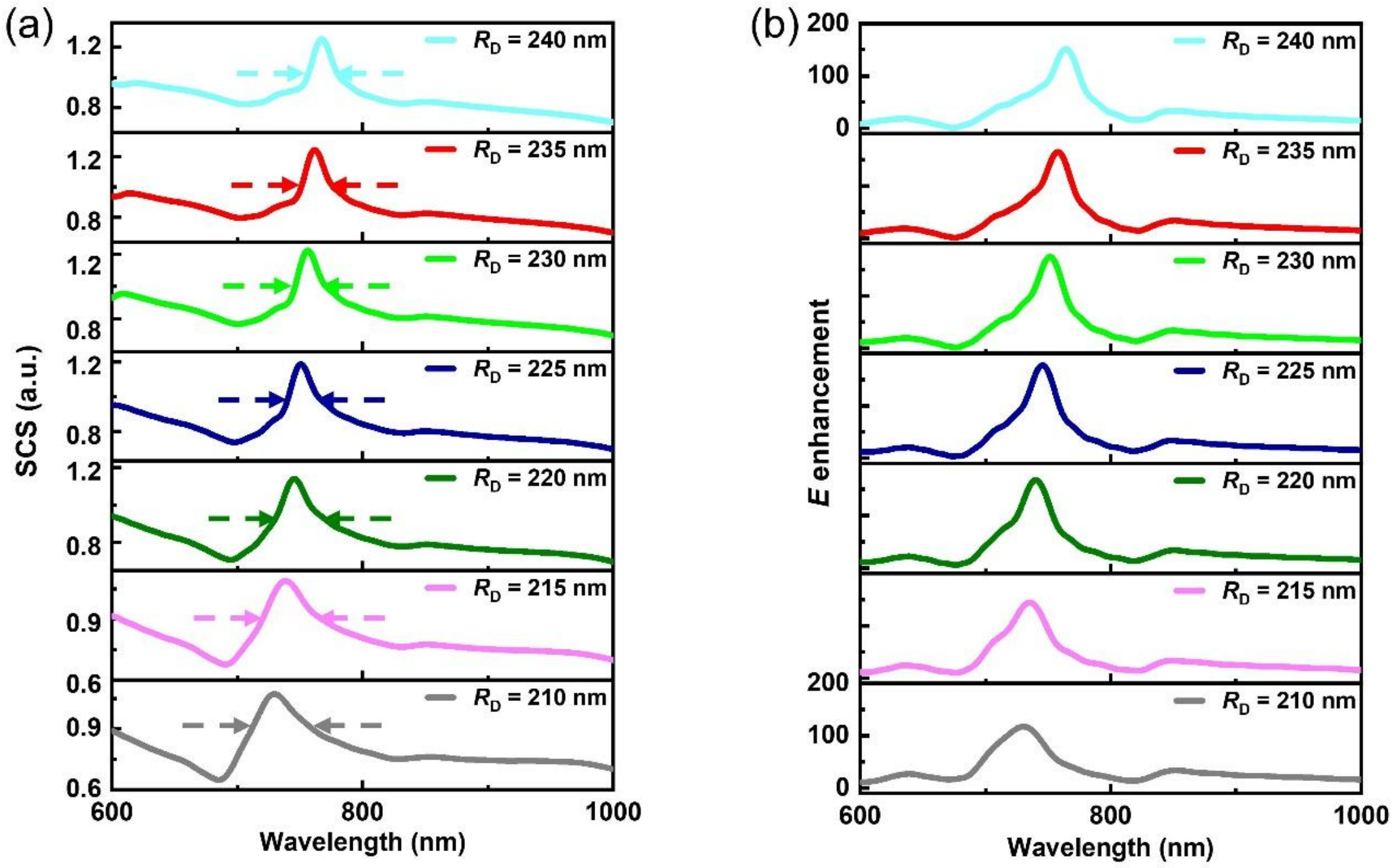


**FIGURE S1.** (a) The far-field spectra of the hybrid cavity as the disk radius varies from $R_D$ = 210 nm to 240 nm. (b) Maximum electric field enhancement spectra of hybrid cavities at varying disk radii.

**Figure S2. Analysis results of higher-order modes in the hybrid cavity.**

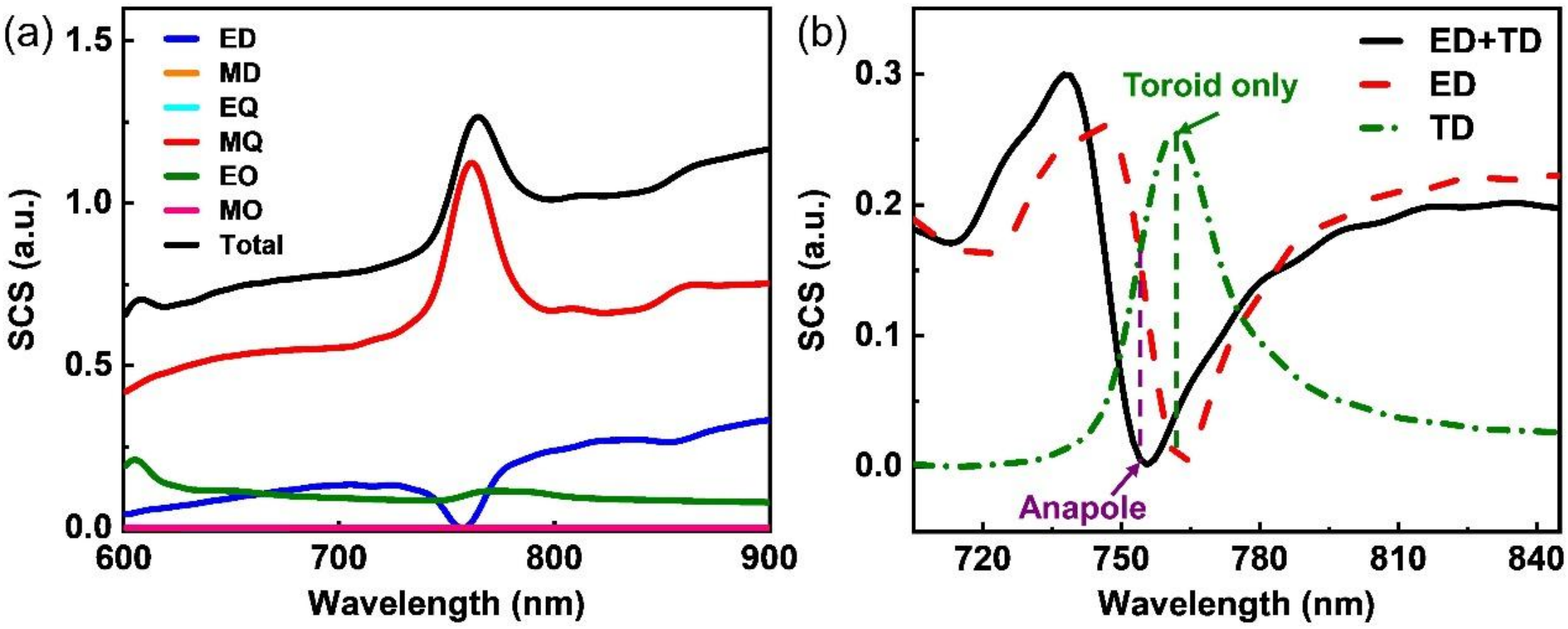


**FIGURE S2.** (a) Cartesian multipole decomposition results of the scattering spectrum for the Si disk with radius $R_D$ = 230 nm. The ED, MD, EQ, MQ, EO and MO represent electric dipole moment, magnetic dipole moment, electric quadrupole moment, magnetic quadrupole moment, electric octupole moment and magnetic octupole moment, respectively. For details on the multipole expression, refer to the Reference [11]. (b) The black curve (ED+TD) represents the anapole state, whose far-field radiation results from the interference between ED and TD.

**Figure S3. Absorption cross section spectrum maps for the two heterostructures.**

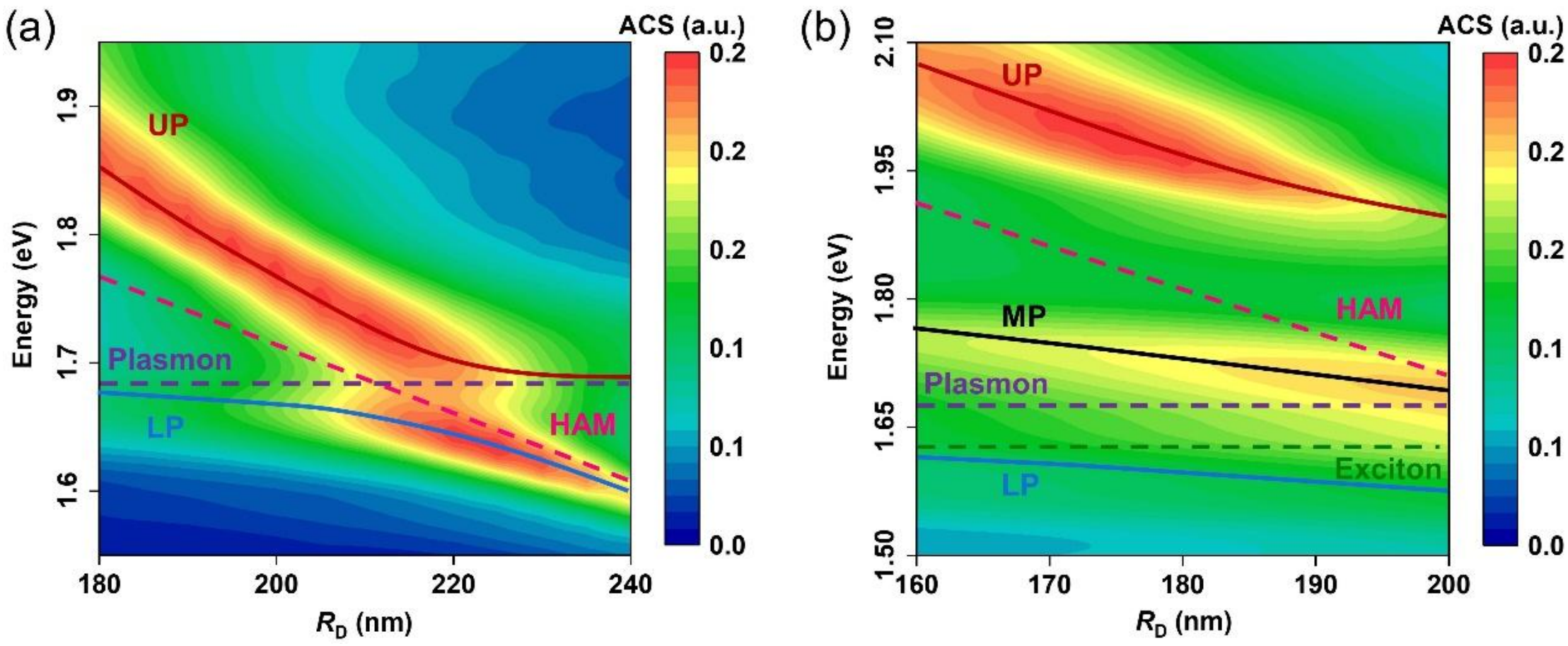


**FIGURE S3.** The absorption cross section (ACS) spectra of the HAS-plasmon (a) and strengthened Si−Au−$WSe_2$ (b) systems as a function of the disk radius $R_D$ (color bar).

**Table S1. Comparison of Rabi Splitting ($\hbar\Omega$) Energy and Normalized Coupling Metric ($\hbar\Omega/\sqrt{N_{eff}}$) in TMDCs**

| **Exciton types** | **Cavity systems** | **$\hbar\Omega$ (meV)** | **$V_{TMD}$ ($nm^3$)** | **$N_{eff}$ ($\times 10^3$)** | **$\hbar\Omega/\sqrt{N_{eff}}$ (meV)** | **Exp./ Theory** | **Ref.** |
|---|---|---|---|---|---|---|---|
| Monolayer $WS_2$ | Magnetic dipole (MD) | 116 | $4.95\times10^4$ | ~11.8 | ~1.068 | Exp. | [12] |
| Bulk $WS_2$ | Anapole | 190 | $2.92\times10^6$ | ~697 | ~0.228 | Exp. | [13] |
| Monolayer $WS_2$ | FP cavity-plasmon | 300 | $5.29\times10^4$ | ~12.6 | ~2.673 | Exp. | [9] |
| Monolayer $WS_2$ | FP cavity-plasmon | 500 | $4\times10^4$ | ~27.8 | ~2.99 | Exp. | [14] |
| Monolayer $WSe_2$ | Anapole | 159 | $1.26\times10^5$ | ~30.1 | ~0.916 | Theory | [15] |
| Monolayer $WSe_2$ | Q-BIC | 29 | $6.15\times10^4$ | ~14.7 | ~0.239 | Theory | [16] |
| Monolayer $WS_2$ | Mirror-image-induced MD | 116 | $1.08\times10^5$ | ~25.8 | ~0.722 | Exp. | [17] |
| Monolayer $WS_2$ | Mirror-image-induced MD | 240 | $9.62\times10^4$ | ~23 | ~1.583 | Exp. | [7] |
| Bulk $WS_2$ | Anapole | 151 | $5.5\times10^5$ | ~131 | ~0.417 | Theory | [18] |
| Monolayer $WS_2$ | Q-BIC | 70 | $1.24\times10^5$ | ~29.6 | ~0.407 | Exp. | [19] |
| Bulk $WS_2$ | FP cavity-q-BIC | 240 | $8.84\times10^5$ | ~211 | ~0.522 | Theory | [8] |
| Bulk $WSe_2$ | Q-BIC | 266 | $7.12\times10^6$ | ~1700 | ~0.204 | Theory | [20] |
| Bulk $WS_2$ | Magnetic quadrupole-anapole | 180 | $2.34\times10^6$ | ~558 | ~0.24 | Theory | [21] |
| **Embedded $WSe_2$** | **Hybrid anapole-plasmon** | **330**<br>**164** | **$2.6\times10^4$**<br>**$1.3\times10^4$** | **~6.2**<br>**~3.1** | **~4.191**<br>**~2.946** | Theory | **This work** |

## Reference

1. S.-D. Liu, J.-L. Fan, W.-J. Wang, J.-D. Chen, and Z.-H. Chen, "Resonance coupling between molecular excitons and nonradiating anapole modes in silicon nanodisk-J-aggregate heterostructures," *ACS Photon.* **5** (2018): 1628-1639.

2. Y.-H. Deng, Z.-J. Yang, M.-L. Hu, X.-J. Du, and J. He, "Boosting an anapole mode response through electromagnetic interactions beyond near-field limit in individual all-dielectric disk-ring nanostructures,"

*New J. Phys.* **23** (2021): 023004.
3. R. Alaee, C. Rockstuhl, and I. J. O. C. Fernandez-Corbaton, "An electromagnetic multipole expansion beyond the long-wavelength approximation," *Opt. Commun.* **407** (2018): 17-21.
4. J. Sun, H. Hu, D. Zheng, D. Zhang, Q. Deng, S. Zhang, and H. Xu, "Light-emitting plexciton: exploiting plasmon-exciton interaction in the intermediate coupling regime," *ACS Nano* **12** (2018): 10393-10402.
5. R. Du, H. Hu, T. Fu, Z. Shi, S. Zhang, and H. Xu, "How to obtain the correct Rabi splitting in a subwavelength interacting system," *Nano Lett.* **23** (2023): 444-450.
6. Y.-H. Deng, Y.-W. Lu, H.-J. Zhang, Z.-H. Shi, Z.-K. Zhou, and X.-H. Wang, "Strong light–matter interactions based on excitons and the abnormal all-dielectric anapole mode with both large field enhancement and low loss," *Photon. Res.* **12** (2024): 854-864.
7. F. Deng, H. Huang, J.-D. Chen, S. Liu, H. Pang, X. He, and S. Lan, "Greatly Enhanced Plasmon–Exciton Coupling in Si/$WS_2$/Au Nanocavities," *Nano Lett.* **22** (2022): 220-228.
8. P. Xie, Q. Ding, Z. Liang, S. Shen, L. Yue, H. Zhang, and W. Wang, "Cavity-assisted boosting of self-hybridization between excitons and photonic bound states in the continuum in multilayers of transition metal dichalcogenides," *Phys. Rev. B* **107** (2023): 075415.
9. B. Li, S. Zu, Z. Zhang, L. Zheng, Q. Jiang, B. Du, Y. Luo, Y. Gong, Y. Zhang, F. Lin, B. Shen, X. Zhu, P. M. Ajayan, and Z. Fang, "Large Rabi splitting obtained in Ag-$WS_2$ strong-coupling heterostructure with optical microcavity at room temperature," *Opto-Electron. Adv.* **2** (2019): 19000801.
10. D. Y. Qiu, F. H. da Jornada, and S. G. Louie, "Optical Spectrum of $MoS_2$: Many-Body Effects and Diversity of Exciton States," *Phys. Rev. Lett.* **111** (2013): 216805.
11. P. Grahn, A. Shevchenko, and M. Kaivola, "Electromagnetic multipole theory for optical nanomaterials," *New J. Phys.* **14** (2012): 093033.
12. S. Lepeshov, M. Wang, A. Krasnok, O. Kotov, T. Zhang, H. Liu, T. Jiang, B. Korgel, M. Terrones, Y. Zheng, and A. Alu, "Tunable resonance coupling in single Si nanoparticle-monolayer $WS_2$ structures," *ACS Appl. Mater. Interfaces* **10** (2018): 16690-16697.
13. R. Verre, D. G. Baranov, B. Munkhbat, J. Cuadra, M. Käll, and T. J. N. n. Shegai, "Transition metal dichalcogenide nanodisks as high-index dielectric Mie nanoresonators," *Nat. Nanotechnol.* **14** (2019): 679-683.
14. A. Bisht, J. Cuadra, M. Wersall, A. Canales, T. J. Antosiewicz, and T. Shegai, "Collective Strong Light-Matter Coupling in Hierarchical Microcavity-Plasmon-Exciton Systems," *Nano Lett.* **19** (2019): 189-196.
15. K. As'ham, I. Al-Ani, L. Huang, A. E. Miroshnichenko, and H. T. Hattori, "Boosting strong coupling in a hybrid $WSe_2$ monolayer-anapole-plasmon system," *ACS Photon.* **8** (2021): 489-496.
16. M. Qin, J. Duan, S. Xiao, W. Liu, T. Yu, T. Wang, and Q. Liao, "Manipulating strong coupling between exciton and quasibound states in the continuum resonance," *Phys. Rev. B* **105** (2022): 195425.
17. S. Liu, F. Deng, W. Zhuang, X. He, H. Huang, J.-D. Chen, H. Pang, and S. Lan, "Optical introduction and manipulation of plasmon-exciton-trion coupling in a Si/$WS_2$/Au nanocavity," *ACS Nano* **16** (2022): 14390-14401.
18. J. Wang, W. Yang, G. Sun, Y. He, P. Ren, and Z. Yang, "Boosting anapole-exciton strong coupling in all-dielectric heterostructures," *Photon. Res.* **10** (2022): 1744-1753.
19. E. Maggiolini, L. Polimeno, F. Todisco, A. Di Renzo, B. Han, M. De Giorgi, V. Ardizzone, C. Schneider, R. Mastria, A. Cannavale, M. Pugliese, L. De Marco, A. Rizzo, V. Maiorano, G. Gigli, D. Gerace, D. Sanvitto, and D. Ballarini, "Strongly enhanced light–matter coupling of monolayer $WS_2$ from

a bound state in the continuum," *Nat. Mater.* **22** (2023): 964-969.
20. L. P. Fan, S. J. You, C. G. Gao, Y. Y. Yu, and C. B. Zhou, "Large Rabi splitting energy in resonant quasi-BIC $WSe_2$ metasurfaces," *J. Phys. D: Appl. Phys.* **57** (2024): 235102.
21. S. Shen, P. Xie, Q. Ding, L. Yue, J. Du, and W. Wang, "Bulk transition metal dichalcogenide disk-ring nanoresonators for intrinsic strong light-matter interaction," *Phys. Rev. B* **111** (2025): 155429.
